# Emotion Analysis of Personalized Color of Beauty Package Based on NCD Image Positioning

**DOI:** 10.3389/fpsyg.2021.727788

**Published:** 2021-08-05

**Authors:** Feng Wang, Jifeng Xu, Hanning Zhang, Jun Yin

**Affiliations:** ^1^School of Design, Jiangnan University, Wuxi, China; ^2^School of Art, Southeast University, Nanjing, China

**Keywords:** NCD image positioning, color emotion analysis, beauty package, color system, color semantics

## Abstract

**Purpose:** Based on the Nippon Color & Design (NCD) system, this study aims to accurately discover the color preferences and image positioning of different female groups in China and, thus, to establish a color reference system, which is suitable for the packaging of beauty products.

**Methods:** We first selected middle-aged women in the rural area of northern China as the studied group. Then, we conducted an extensive questionnaire survey on their color preferences focusing on their psychological characteristics and perception rules of emotional needs for colors. After that, we extracted colors from samples and classified them using the NCD system research method. Finally, we conducted a systematic analysis and verification to determine the color emotional preference of this group.

**Conclusion:** We investigate the color preference of targeted customers to establish the image space of beauty product packaging. By applying SPSS for the factor analysis, we study the rural women in northern China, who are less concerned about fashion. We focus on the accurate positioning between the interaction of color, women, and environment throughout the color design process. We have conducted a series of comparative analyses of popular beauty products among the rural women. The results can accurately reflect the brand definition and positioning of color semantics and, thus, provide invaluable information for future beauty packaging designs and marketing promotion strategy.

## Introduction

Due to the rapid development of the economy of China in the past two decades, the per capita disposable income of Chinese residents has been growing swiftly, which has led to a substantial increase in the beauty product market. According to the report of China's National Bureau of Statistics in 2020, the sales in the cosmetics market of China have increased from 204.9 billion yuan in 2015 to 340 billion yuan in 2020. Currently, China is the second largest cosmetics consumption country in the world after the United States. In order to compete in the fast-growing market, many researchers start to study the purchasing behaviors of customers and their psychological states. How to lure customers to purchase their products becomes their top priority. Hence, emotional designs have become the primary concern in the design industry.

According to the survey shown in *A study about package design and color elements of digital products* by Koh, Seon, the colors of the product packaging design have a great impact on the purchase desires of consumers; 78% of the survey participants said the color would affect their purchase desires. The outcome of the survey shows that the color of the product packaging design is essential to sales. As the primary element in visual impression, the color of packaging design directly affects the physiological response and psychological cognition of people. Relevant theoretical foundations include, for example, *A Study of Color Emotion and Color Preference*. In this study, three color preference models for single colors were developed (Ou et al., 2004), *Atypical Color Preference in Children with Autism Spectrum Disorder* (Marine and Nobuo, 2016), *Color Preference and Food Choice among Children* (Walsh et al., 1990), and *Relations between Their Color Preference and Colors of Their Clothes Color Preference of Little Children* (Haruko and Emiko, 1972).

Color emotion is a psychological reaction that associates with the long-term understanding of people of objective things and their related colors, and it manifests itself as the emotional expression of color and the emotional experience brought by color. According to the survey shown in *Color Preferences in Adolescence and Early Adulthood and Their Relationship with Emotional Intelligence* by Bolshunova and Khromova (2021), the article is to identify the age specificity of color preferences and their connection with some personality traits presented in the parameters of emotional intelligence in adolescence and early adulthood. In *Seasonal Variations in Color Preference* by Schloss et al. (2017), empirical support for this distinction shows that pair preference and harmony both increase as hue similarity increases, but preference relies more strongly on component color preference and lightness contrast (Schloss and Palmer, 2011).

Color itself can express certain moods and emotions. There are many factors affecting color psychology. The psychological association and emotional representation of color are multifaceted and complex. First, color perception is closely related to age, occupation, and gender. In *Comparative Studies on Color Preference in Japan and Other Asian Regions, with Special Emphasis on the Preference for White* (Saito, 1996), factor analysis and cluster analysis indicated some relationship between color preference and the lifestyles of subjects. Besides the factors of age and sex, associative images based on environmental and cultural aspects may be an important factor influencing color preference. Second, the application of color in different situations will bring people different emotional experiences. Finally, the symbolic meanings of colors in different countries, regions, and nations are also different. When designing products, we must fully understand the influencing factors of color psychology in order to correctly grasp the application rules of color emotion in the design field. We have to carry out color collection, summarization, and symbolic expression, establish the expression of color intentions, and incorporate them into the color design process.

Of the 1.4 billion people in China, 40.42% are farmers, and female employees account for 43.7% of total employees (Sin et al., 2001). Northern China is a traditional agricultural province, with Qinling Mountains and Huaihe River as the dividing line between the northern and southern regions. Northern women are deeply influenced by traditional culture and customs, especially rural women who are the typical representative of traditional Chinese women. Judging from the composite consumption index of China in recent years, the female consumer market in China occupies an increasingly important position (Tam and Tai, 1998).

The research objective of this study is aimed at the minority group of Chinese women who are ignored by the present designers and the market. They are traditional conservative women with introverted personality and low educational level in the northern rural areas. In this study, the research on color emotional needs of this group can reflect the problem awareness of the dynamic development of the cosmetic market and, thus, highlight the research and practical value of this topic.

### NCD Color Image Coordinate System

The NCD semantic positioning system was developed by the Japan Institute of Color Design, which is based on the theories of perceptual engineering and color psychology, and established through experimental parameters reflecting the mapping relationship between color and semantics. This color matching system provides a valuable reference for modern design practices. The color psychologist Kobayashi Shigshun established the NCD color system through more than 40 years of research studies. He systematically categorized the meaning of color from the image perspective on the basis of the principle of western color science as well as through the basic platform of sociology, iconography, and psychology, and, thus, constructed a system that is suitable for local human environment and more in line with modern design concepts. According to the survey shown in *a study of color emotion and color preference. Part i: color emotions for single colors* by Ou et al. (2010), this article classifies color emotions for single colors and develops science-based color emotion models. The factor analysis identified three color-emotion factors: color activity, color weight, and color heat. The three factors agreed well with those found by Kobayashi and Sato et al. Four color-emotion models were developed, namely, warm–cool, heavy–light, active–passive, and hard–soft.

**Figure 1:**
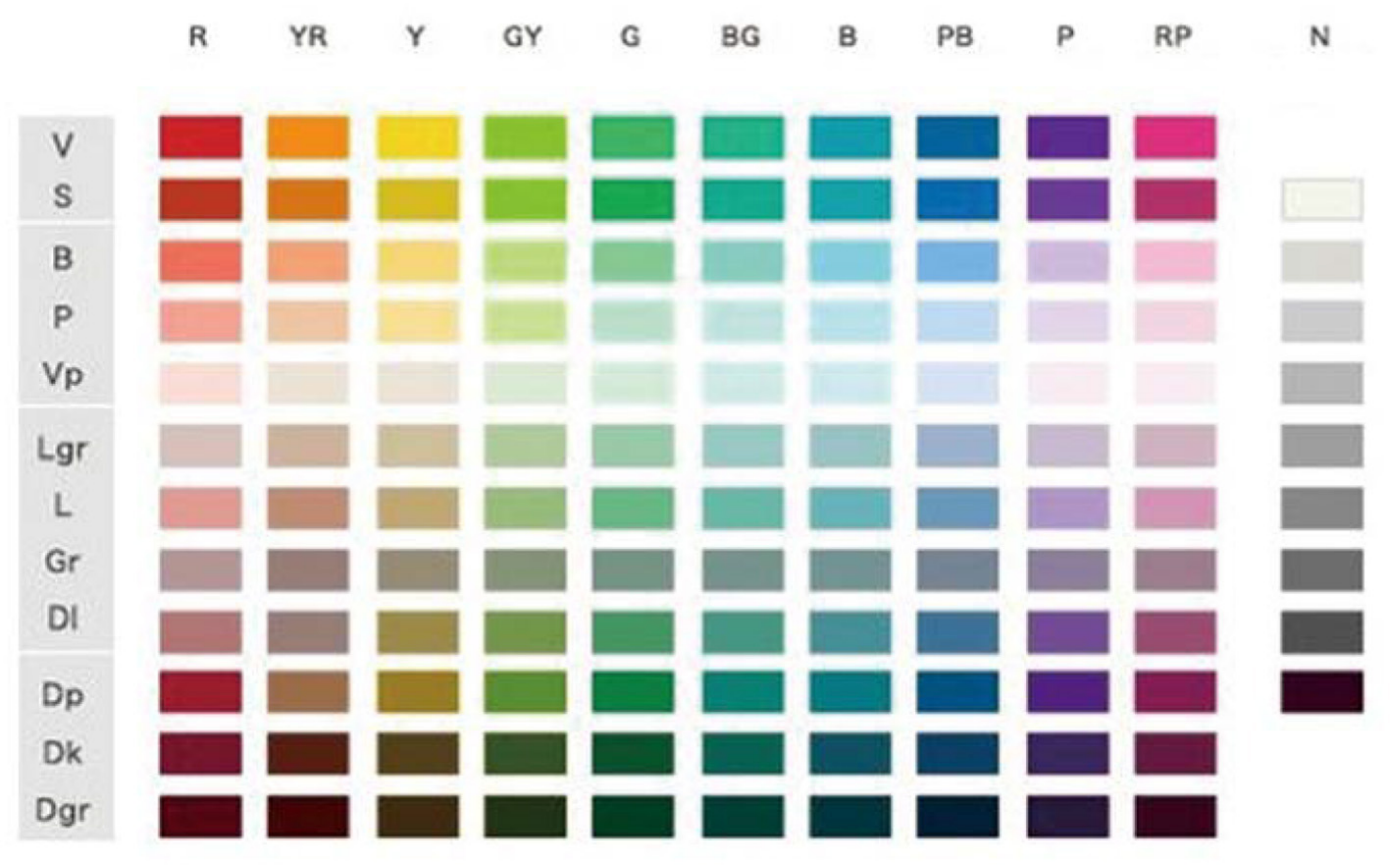
Hue and tone system.

**Figure 2:**
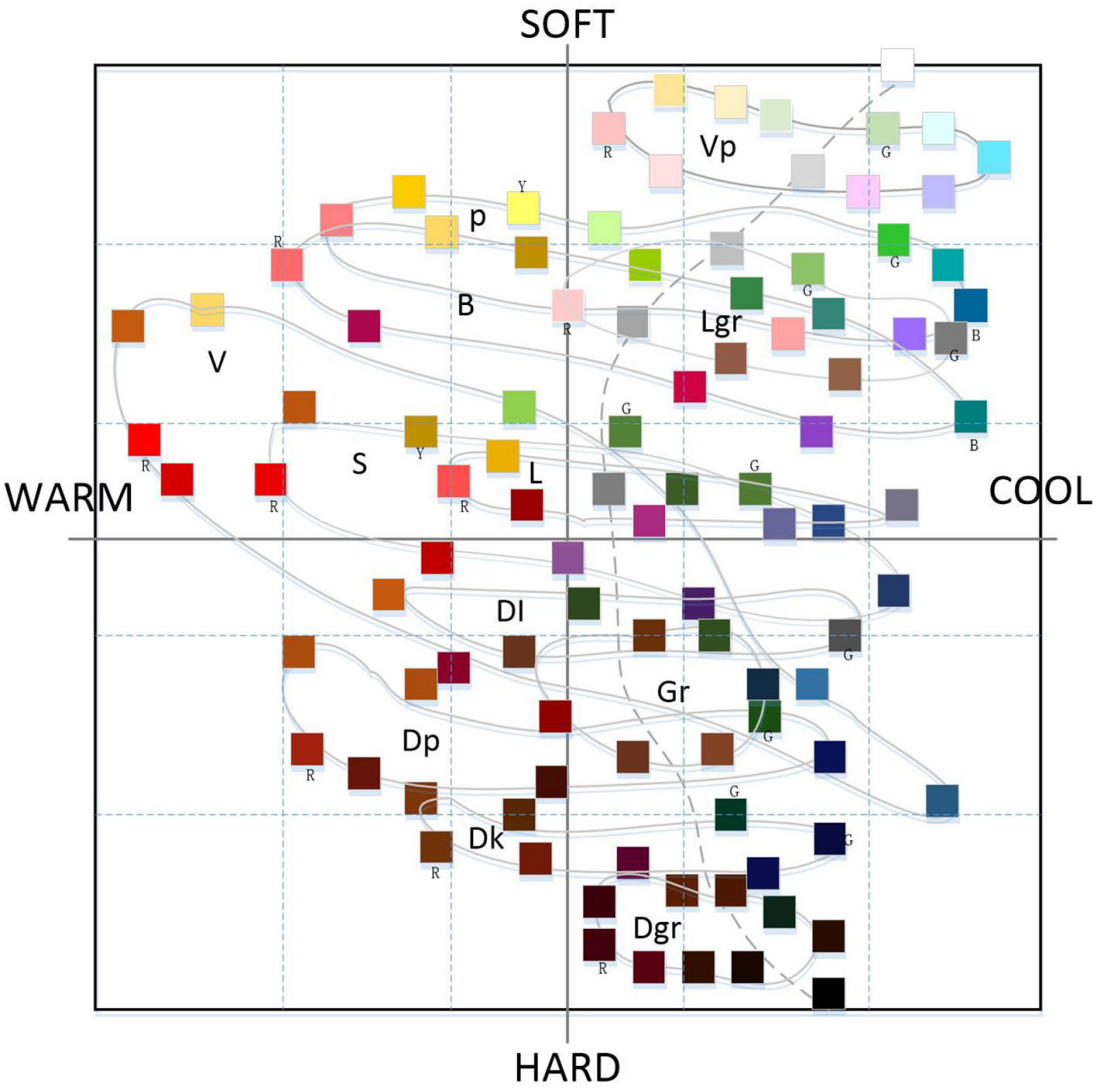
Monochrome image coordinates.

The NCD semantic system is a positioning system that innovatively establishes emotional semantic words based on a variety of sensory experiences of different users. Through rational design logic thinking, the image space between color and perceptual vocabulary is constructed by three psychological axes of cold and warm axis (W–C), soft and hard axis (S–H), and clear turbidity (K–G), and the image system is established on this basis. Figure 1 shows the hue and tone system. There are 120 types of colors arranged in the horizontal and vertical orders according to the hue, and 10 types of chromatic colors, totally 130 types of colors. Figure 2 shows the monochrome image coordinates, which are made according to the data obtained from the psychological survey of language sense, and the colors of the same tone are connected by lines.

### Color Positioning of Beauty Packaging at Home and Abroad

The NCD semantic system is color-matching of a semantic coordinate system, which is formed by analyzing the perspective of color image based on the three basic attributes of color (hue, brightness, purity). The NCD semantic system converts the perceptual psychological needs of users, such as sensory impressions and aesthetic preferences, into semantic symbols, matches the design of cold, warm, soft, and hard colors to meet the perceptual needs of users, and finally proposes solutions to meet the color emotional preferences of different users.

This research investigates, analyzes, and classifies the color status of beauty packaging at home and abroad. We try to explore an accurate and effective color design method to design the color of beauty packaging to enhance the emotional experience of customers. Combining the research theme of color imagery, the practical color coordinate system (PCCS) is used to refine and abstract the color of selected beauty packaging samples, and to conduct statistical and visual analysis of color distribution and visualization of the color style positioning and emotional tendency of beauty brands at home and abroad. Based on the NCD semantic system, its essence is to achieve the consistency of the mapping between color semantics and user perception on the premise that color image positioning is consistent with the perceptual needs of the user. A consistent quantitative basis is established, and the perceptual engineering and quantitative color systems is applied to complete the quantitative research of matching objects at both ends of the mapping, which provides the feasibility for establishing consistency in orientation.

1) The hue and tone distribution of beauty packaging at home and abroad

After preliminary investigations, we selected basic skin care products as the subject of this research. Contemporary women are very demanding on their own appearances, 78% of women are not satisfied with their skin conditions, and their requirements for beauty are getting higher and higher. Among them, anti-aging has become their main demand. CBNData research shows that from the consumption of different effects in the online female skin care industry in 2020, the demand for anti-aging skin care accounted for 34%, exceeding the long-term concern of most women for moisturizing and moisturizing needs (33.4%). The significance of choosing this type of beauty product research lies in three aspects. First, basic skin care products are the most representative products in the current beauty industry. Second, the purchase frequency of skin care products is relatively fixed, and their seasonal characteristics are strong. The main purchases are quarterly basis, with one to two times a quarter being the mainstream purchase frequency. Third, the consumption level of skin care products tends to be even, and there are a considerable number of consumer groups in the middle, high, and low categories.

**Figure 3:**
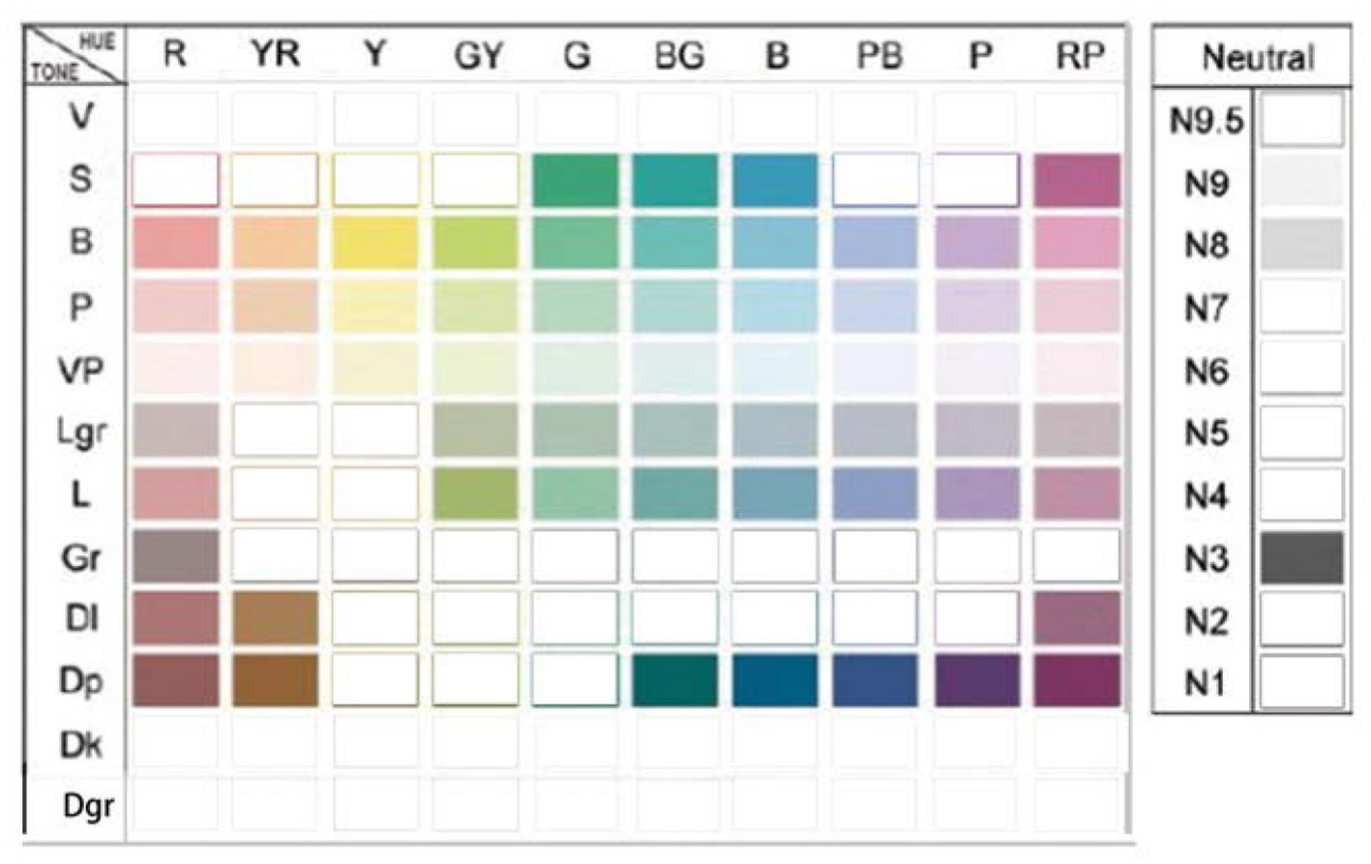
Hue and tone distribution of cosmetics packaging in China and abroad.

The survey collected 200 samples of beauty packaging at home and abroad. The main containers and packaging of skin care products are usually plastic bottles, glass bottles, hoses, and airless bottles. Factors such as different painting processes and reflective surfaces of the outer packaging are excluded, and the final color application situation is shown in Figure 3. The survey results show that the overall hue of basic skin care products is more blue-green than red-orange. This aspect is related to the use of the product by a consumer, mainly for cleansing, moisturizing, and moisturizing. The color is cooler, giving a refreshing and clean feeling. From the perspective of different origins and brand image positioning, achromatic gray and white are used more frequently in the packaging of European and American skin care products. In addition, cool hues such as blue-green and blue appear more frequently. Japanese and Korean brands tend to have warm red-orange colors, which are softer and warmer than European and American brands. The colors of Southeast Asian brands tend to be more gorgeous, the colors of domestic Chinese brands are more diverse, and product segmentation is closer to the specific needs of consumers.

2) Color distribution of cosmetics packaging in image space

**Figure 4:**
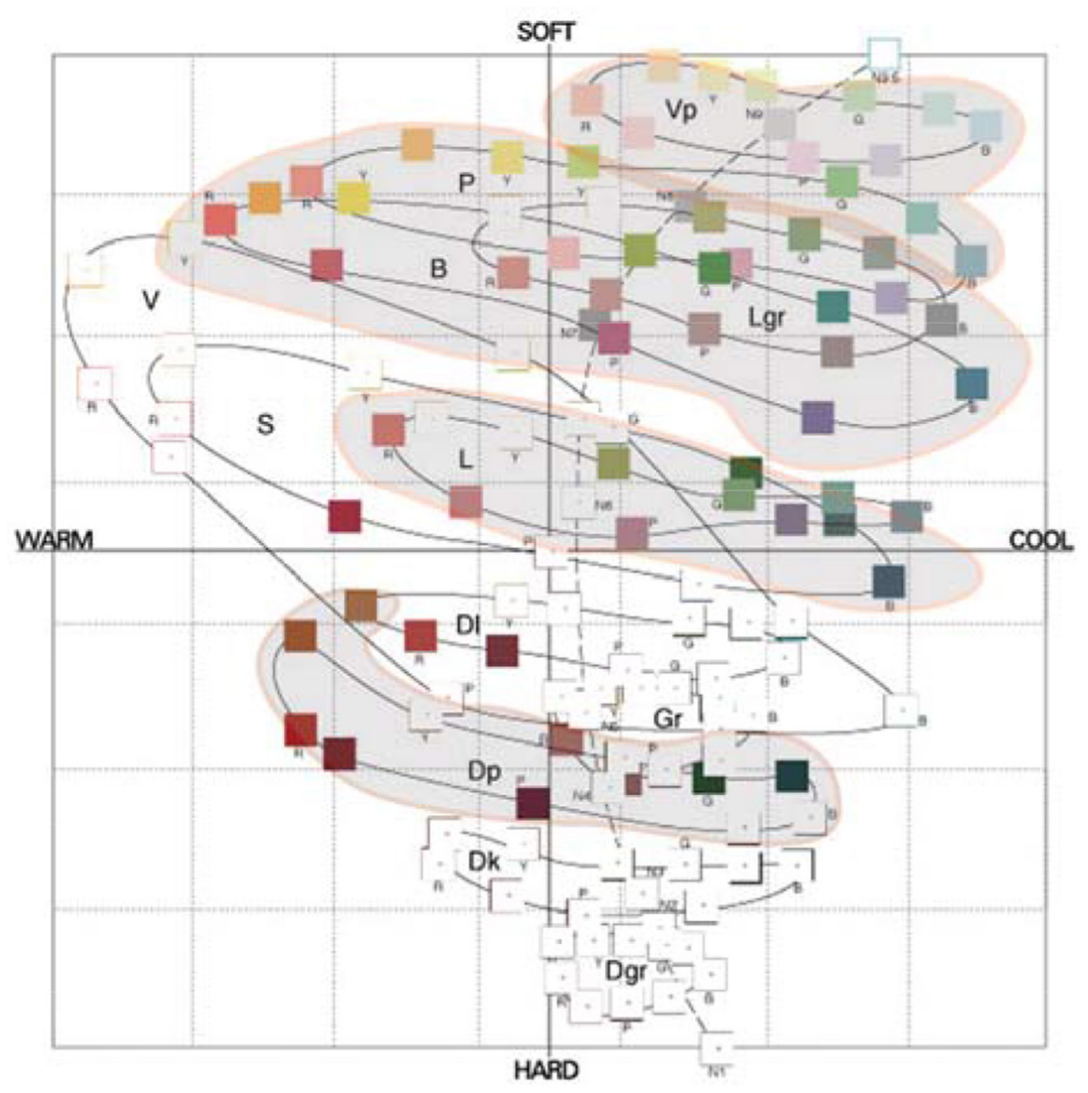
Color distribution of cosmetic packaging in image space.

The distribution of the packaging colors of basic skin care products at home and abroad in the image space is shown in Figure 4. The distribution is mainly concentrated on the upper right of the color arc, showing a tendency to be colder overall, with relatively more cool colors. The color group below the central axis, the low-brightness dark color group, is obviously less than the high-brightness color group above the central axis. The lightness and purity of the two ends of the axis are relatively more tones. The color is relatively soft in terms of softness and hardness, and the soft colors are more concentrated, indicating that the main color and light color have the same lightness and, thus, the emotional demand for the product is more consistent.

## Color Emotion Analysis of Middle-Aged Women in Rural Areas of Northern China on Beauty Packaging

### Extraction of Representative Semantics of Beauty Packaging Color Image

This research is based on the collection and selection of color image adjective pairings of beauty packaging. First, after determination of the representative color test samples of beauty packaging in the previous steps (Figures 3, 4), we will explore the color design and planning process of beauty packaging. In order to gain more comprehensive and accurate image cognition of testing the beauty packaging, all the colors of the beauty packaging were paired together, and then an integral color image of this product series was formed. The color positioning and emotional semantics produced by this overall image are relatively clear, rich, and perceptible. The main references include: Kobayashi Shigshun, Analysis of Color Psychology; the website “NIPPON COLOR & DESIGN RESEARCH INSTITUTE”; Fujisawa Hideaki, Color psychology; ChiQianYanHideaki, The Incredible Psychological Colors; Tadashi Oyama, The Psychology of Color; Heller & Wu Tong, and The Character of Color. Among them, 180 adjectives commonly used in color image analysis summarized by Japan Color Research Institute are involved.

On this basis, we perform a detailed analysis of the specific meaning of each group of adjectives, and deleted the adjective groups with similar meanings, similarities, and unclear expressions. We also search relevant literature, referring to the cognitive suggestions of cosmetic design practitioners on the color of beauty packaging, and then obtain the semantic description related to the color image of beauty packaging. Finally, we select 30 sets of color image adjective pairs as the final test vocabulary, as shown in Table 1.

**Table 1:** Selected 30 sets of color image adjective pairs.

1. Emotional–intellectual	11. Youthful–mellow	21. Intellectual–progressive
2. Elegant–dandy	12. Modern–traditional	22. Complex–simple
3. Sweet–bitter	13. Stylish–classic	23. Abundant-rich–pure
4. Masculine–feminine	14. Lighthearted–intense	24. Natural–artificial
5. Casual–formal	15. Provocative–sober	25. Alluring–conservative
6. Dynamic–calm	16. Agile–sedate	26. Provincial–urban
7. Gorgeous–plain	17. Speedy–smooth	27. Luxurious–rustic
8. Lighthearted–nostalgic	18. Fascinating–rustic	28. Romantic–practical
9. Fashionable–old-fashioned	19. Passionate–grave	29. Negative–positive
10. Western–eastern	20. Innocent–elaborate	30. Delicate–coarse

### Determine Middle-Aged Women in Northern Rural Areas of China as the Research Object

The female consumer market in China is huge, and the rapidly growing market has led to changes in the market structure. The growth of female users will stimulate the overall market. Exquisite product packaging and unique emotional experience often tend to arouse the consumer psychology of this group and stimulate consumer impulse. Therefore, studying the color preferences of female consumer groups has great potential and value for the market. White collar workers are the mainstream consumers of skin care products. They will choose first- and second-tier mid- to high-end products. As far as the development of domestic brands is concerned, the market value of domestic brands is that they are better at developing channels in second- and third-tier cities than international brands, and quickly seize the mass market in second- and third-tier cities and rural areas.

In this case, the middle-aged female consumer group of about 30–55 years old is selected as the study subject. There are three main reasons for studying the northern rural women of this age group:

1. The consumption behaviors of northern rural women under the age of 30 have not yet been fixed, and they are mostly seeking differentiation and have a strong sense of group imitation consumption;
2. Among the northern rural woman consumers who are over 55 years of age, consumption behaviors and consumption habits have reached a fixed pattern in the middle and young ages, and their personal purchasing intentions have weakened, and new consumer products will be purchased by their children;
3. Among the northern rural women, those who are 30–55 years of age have a leading role in daily commodity decision-making and purchasing activities. They are not only the decision-makers and purchasers of their own consumption activities but also of their husbands, children, and parents. Due to the influence of different consumer demand, factors such as different personalities, different regions, and different cultural education levels, female consumers in this age group have a certain degree of representativeness. When they purchase cosmetic products, they are more susceptible to the consumer psychology of product appearance and demands for aesthetic emotion.

## Factor Analysis of Color Image Semantics in Beauty Packaging

The method of factor analysis was widely used in semantics analysis research. For example, the study investigates the relationship between color perceptual attributes and color emotions, as well as the influence of different cultural backgrounds. It was concluded that chroma and lightness were the most important factors on color emotion, whereas the influences of hue and cultural background were very limited. In *Analysis of cross-cultural color emotion* by Gao et al. (2010), semantic-differential data of color emotions for color pairs were collected and examined by factor analysis (FA). In *Analyses of Color Emotion for Color Pairs with Independent Component Analysis and Factor Analysis* by Hanada (2013), word associations or verbal synesthesia between concepts of color and emotions were studied in Germany, Mexico, Poland, Russia, and the United States. With emotion words as the between-subjects variable, 661 undergraduates indicated on a 6-point scale to what extent of anger, envy, fear, and jealousy 12 terms of color reminded them in *Correspondence Analysis of Color–Emotion Associations* by Hanada (2018). In *The Color of Music: Correspondence through Emotion* by Barbiere et al. (2007), it was concluded that music-color correspondences occur *via* the underlying emotion common to the two stimuli, and this study investigated the relationship between color perceptual attributes and color emotions, as well as the influence of different cultural backgrounds.

Based on 97 questionnaires, the average value of the semantic evaluation of each color sample is computed. We use SPSS to perform factor analysis, and by the correlation analysis of each variable, the suitability of factor analysis is judged, and the principal component analysis method is used for variable conversion. Using new variables to construct and find several relatively independent new variables can better explain the changes of the original semantic factors, thus constituting a common factor. Then, according to the corresponding variables and samples, the cognitive image space map is drawn to facilitate the formation of color cognition of beauty packaging.

In the first factor analysis, there were a total of 31 factors, and the results showed that there was a high factor load in many factors. Therefore, the topic was deleted and then re-analyzed. After the second factor analysis, the results showed that there was still a high factor load in many factors, so the topic was deleted, and we repeated the analysis.

### Test on the Stability of Factor Analysis

**Figure 5:**
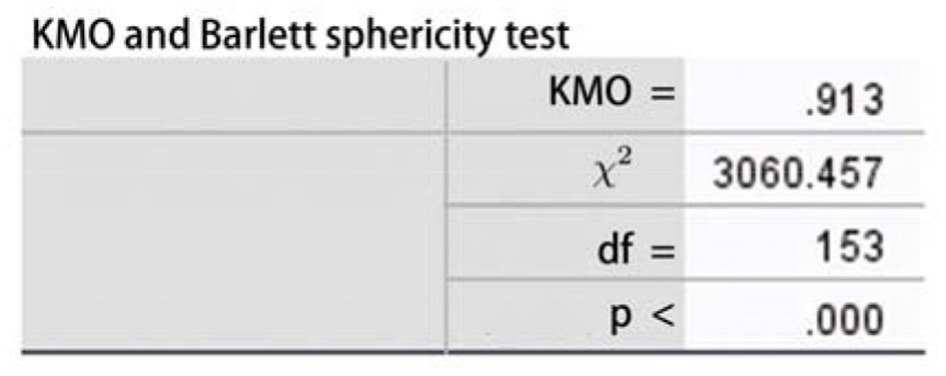
Kaiser–Meyer–Olin (KMO) and Bartlett's test.

**Figure 6:**
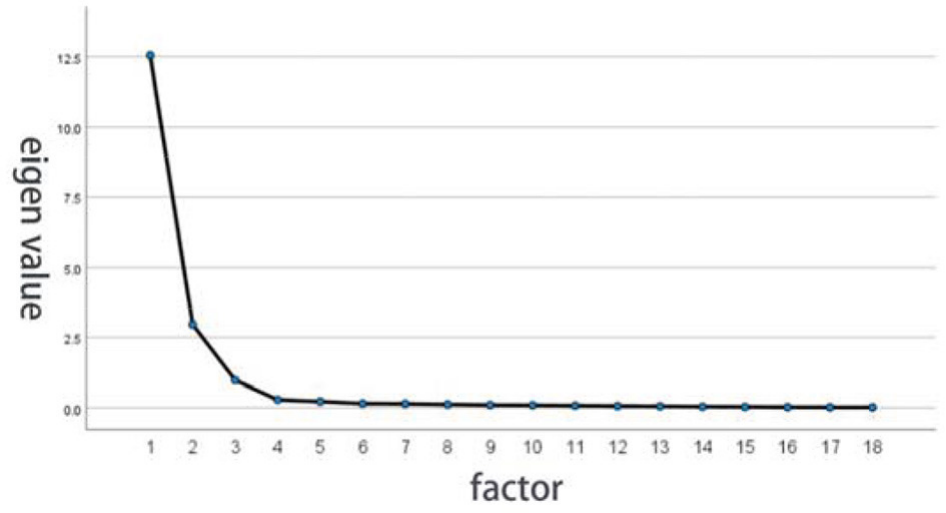
Eigenvalue factor analysis of screen plot.

In order to test whether the original data are suitable for factor analysis, it is necessary to pass the correlation coefficient matrix, Kaiser–Meyer–Olin (KMO) and Bartlett sphericity test, and the test of the common degree of variables. The variants are not irrelevant to each other (*X*^2^ = 3060.475, *df* = 153, *p* < 0.001) and the result shows KMO = 0.913, as shown in Figure 5. According to the measurement standard of KMO provided by Kaise (0.9 < KMO <1, very suitable), the material is suitable for factor analysis. When using the screen plot shown in Figure 6, three factors should be chosen, and these variables are suitable to conduct the factor analysis.

In order to determine the degree of correlation between the extracted common factors and the original variables, it is necessary to test the degree of commonality of the variables. For example, Table 2 shows the common degree of the variable output by the software. The common degree of the first variable “delicate-coarse” in the table is 0.98, indicating that all the extracted factors together explain the 0.98% of the variation information produced by the variable “delicate/coarse.” From the results, the common degree of the 17 variables is >0.8, which means that extracting three common factors is an ideal state.

**Table 2:** Common degrees of variables.

	**Variable**	**Original**	**Extract**
01	Emotional–intellectual	1.000	0.69
02	Elegant–dandy	1.000	0.95
03	Sweet–bitter	1.000	0.86
04	Masculine–feminine	1.000	0.33
05	Casual–formal	1.000	0.47
06	Dynamic–calm	1.000	0.84
07	Gorgeous–plain	1.000	0.89
08	Lighthearted–nostalgic	1.000	0.90
09	Fashionable–old-fashioned	1.000	0.95
10	Western–eastern	1.000	0.87
11	Youthful–mellow	1.000	0.98
12	Modern–traditional	1.000	0.61
13	Stylish–classic	1.000	0.41
14	Lighthearted–intense	1.000	0.95
15	Provocative–sober	1.000	0.83
16	Agile–sedate	1.000	0.97
17	Speedy–smooth	1.000	0.86
18	Fascinating–rustic	1.000	0.87
19	Passionate–grave	1.000	0.59
20	Innocent–elaborate	1.000	0.47
21	Intellectual–progressive	1.000	0.97
22	Complex–simple	1.000	0.46
23	Abundant-rich–pure	1.000	0.32
24	Natural–artificial	1.000	0.94
25	Alluring–conservative	1.000	0.73
26	Provincial–urban	1.000	0.61
27	Luxurious–rustic	1.000	0.98
28	Romantic–practical	1.000	0.66
29	Negative–positive	1.000	0.53
30	Delicate–coarse	1.000	0.98

Three factors are extracted using the principal axis method and carrying out the promax. As shown in Table 3, the element loading martix shows that the first factor contains eight questions, whose contents are mostly related to elegant sense of quality (such as delicate/coarse, intellectual/progressive, fashionable/old-fashioned, lighthearted/intense, elegant/dandy, natural/artificial, lighthearted/nostalgic, and sweet/bitter). The second factor contains six questions, whose contents are mostly related to dynamic sense of youth (such as youthful/mellow, agile/sedate, speedy/smooth, dynamic/calm, provocative/sober, and emotional/intellectual). The third factor contains four questions, whose contents are mostly related to area sense of luxury (such as luxurious/rustic, gorgeous/plain, western/eastern, and fascinating/rustic).

**Table 3:** Pattern matrix diagram.

		**Element**		
		**1**	**2**	**3**
01	Delicate–coarse	0.98		
02	Intellectual–progressive	0.97		
03	Fashionable–old-fashioned	0.95		
04	Lighthearted–intense	0.95		
05	Elegant–dandy	0.95		
06	Natural–artificial	0.94		
07	Lighthearted–nostalgic	0.90		
08	Sweet–bitter	0.86		
09	Youthful–mellow		0.98	
10	Agile–sedate		0.97	
11	Speedy–smooth		0.86	
12	Dynamic–calm		0.84	
13	Provocative–sober		0.83	
14	Emotional–intellectual		0.69	
15	Luxurious–rustic			0.98
16	Gorgeous–plain			0.89
17	Western–eastern			0.87
18	Fascinating–rustic			0.87

### Determination of the Number of Extracted Factors

It can be seen from the output data shown in Table 4 that the eigenvalue of the first factor solution is 12.548, which explains 69.711% of the total variation information of all the 18 variables and is a principal component with the largest variance contribution. The eigenvalue of the fourth factor solution is 0.289. All the eigenvalues are < 1 and, hence, only the first three factors are extracted as common factors. Besides, the first three factors show 91.587% of the total variation information of all variables, reaching a good level. Therefore, it is relatively appropriate to extract three common factors. After orthogonal rotation, the variance contributions of factors are different; the purpose is to increase the load difference of each variable and make the variation in the correlation matrix to be scattered to different factors as far as possible, so the respective contribution rates after rotation are 69.711, 86.149, and 91.587%.

**Table 4:** Explanation of total variance.

**Component**	**Total**	**Original value**	**Cumulative**	**Total**	**Quadratic sum**	**Cumulative**	**Total after rotation**
1	12.548	69.711	69.711	12.548	69.711	69.711	11.065
2	2.959	16.438	86.149	2.959	16.438	86.149	10.973
3	0.979	5.438	91.587	0.979	5.438	91.587	9.045
4	0.289	1.606	93.193				
5	0.228	1.268	94.461				
6	0.157	0.871	95.332				
7	0.148	0.822	96.154				
8	0.125	0.693	96.847				
9	0.105	0.582	96.429				
10	0.096	0.535	97.965				
11	0.082	0.455	98.419				
12	0.069	0.381	98.800				
13	0.059	0.330	99.131				
14	0.046	0.253	99.384				
15	0.038	0.209	99.593				
16	0.029	0.163	99.756				
17	0.024	0.131	99.887				
18	0.020	0.113	100.000				

### Factor Extraction and Naming

Tables 5, 6 show the output factor loading matrix without rotation and the factor loading matrix after rotation, respectively, with the corresponding relationship between the original variables and the common factors. The factor loading matrix without rotation shows that the loads of 18 variables are higher on the first factor. The loads of the variables “delicate/coarse,” “intellectual/progressive” are the highest, 0.99 and 0.98, respectively. The two variables “youthful/mellow” and “agile/sedate” have relatively high loads on the second factor, which are 0.99 and 0.97, respectively. The variable “luxurious/rustic” has the highest load, which is 0.98 on the third factor. Therefore, the variance maximization method is used to further differentiate the size of orthogonal rotation load, so that the corresponding relationship between variables and factors is clearer, and that the main variables affected by each factor can be identified more easily.

**Table 5:** Factor loading matrix without rotation.

		**Element**		
		**1**	**2**	**3**
01	Delicate–coarse	0.99		
02	Intellectual–progressive	0.98		
03	Fashionable–old-fashioned	0.96		
04	Lighthearted–intense	0.95		
05	Elegant–dandy	0.91		
06	Natural–artificial	0.94		
07	Lighthearted–nostalgic	0.87		
08	Sweet–bitter	0.43		
09	Youthful–mellow		0.99	
10	Agile–sedate		0.97	
11	Speedy–smooth		0.87	
12	Dynamic–calm		0.86	
13	Provocative–sober		0.83	
14	Emotional–intellectual		0.70	
15	Luxurious–rustic			0.98
16	Gorgeous–plain			0.88
17	Western–eastern			0.88
18	Fascinating–rustic			0.86

**Table 6:** Factor loading matrix after rotation.

		**Element**		
		**1**	**2**	**3**
01	Delicate–coarse	0.98		
02	Intellectual–progressive	0.97		
03	Fashionable–old-fashioned	0.95		
04	Lighthearted–intense	0.95		
05	Elegant–dandy	0.95		
06	Natural–artificial	0.94		
07	Lighthearted–nostalgic	0.90		
08	Sweet–bitter	0.86		
09	Youthful–mellow		0.98	
10	Agile–sedate		0.97	
11	Speedy–smooth		0.86	
12	Dynamic–calm		0.84	
13	Provocative–sober		0.83	
14	Emotional–intellectual		0.69	
15	Luxurious–rustic			0.98
16	Gorgeous–plain			0.89
17	Western–eastern			0.87
18	Fascinating–rustic			0.87

The first factor mainly affects three groups of variables: delicate/coarse, intellectual/progressive, and fashionable/old-fashioned, among which the load of delicate/coarse is the highest, which is 0.98. These three groups mainly describe the rational cognition of color images, which can be called “cognitive factors.”

The second factor mainly affects three groups of variables: youthful/mellow, agile/sedate, and speedy/smooth, among which the load of youthful/mellow is the highest, which is 0.98. These three groups are mainly about direct experience and feelings of color images, which can be called “activity factors.”

The third factor mainly affects four groups of variables: luxurious/rustic, gorgeous /plain, western/eastern, and fascinating/rustic, among which the load of luxurious/rustic is the highest, which is 0.98. These four groups mainly describe the social and physical attribution of color, which can be called “attributive factors.”

### The Corresponding Relation Between Sample Colors and Factor Axis

**Figure 7:**
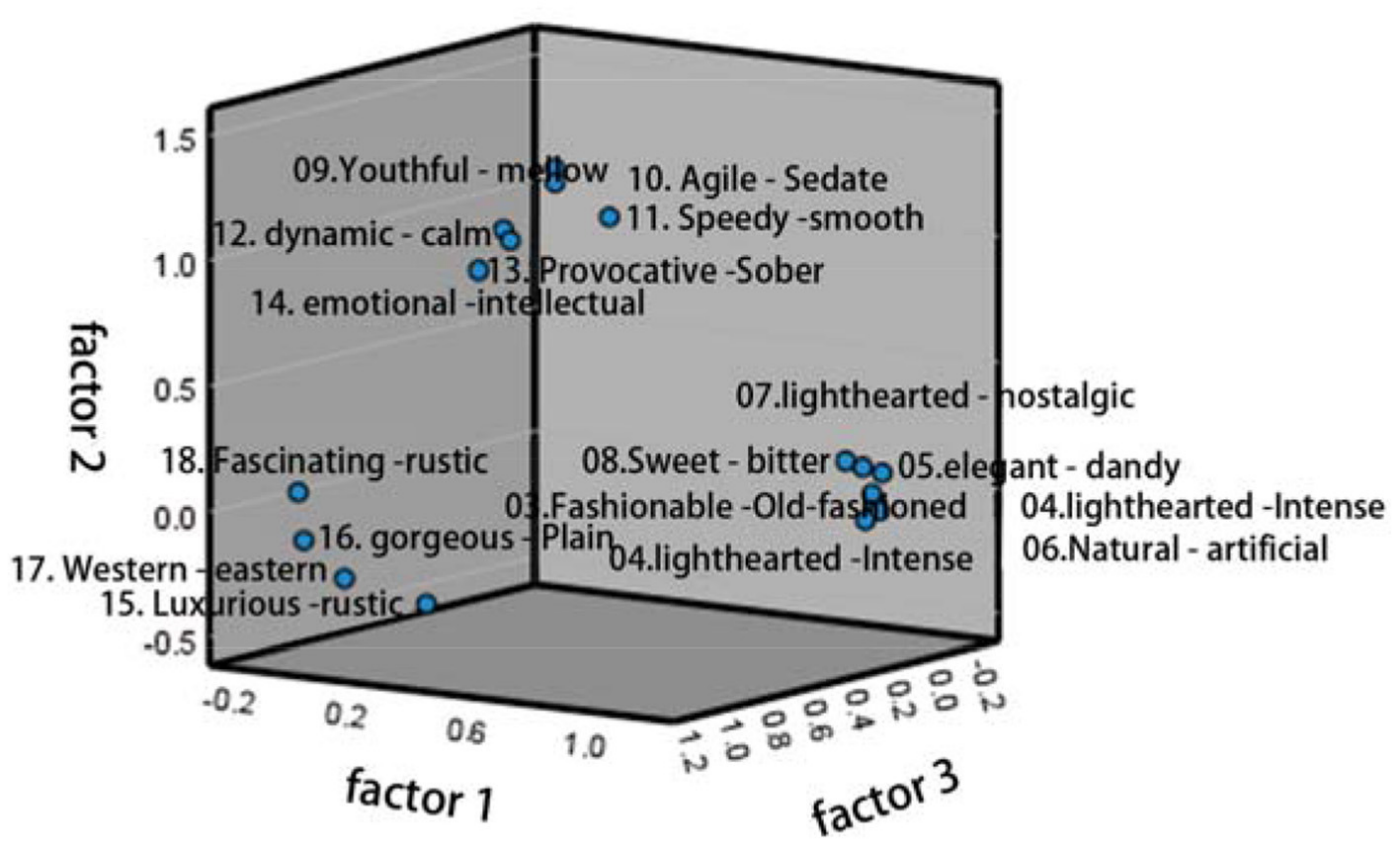
Distribution of color semantics in the three-dimensional cognitive space.

**Figure 8:**
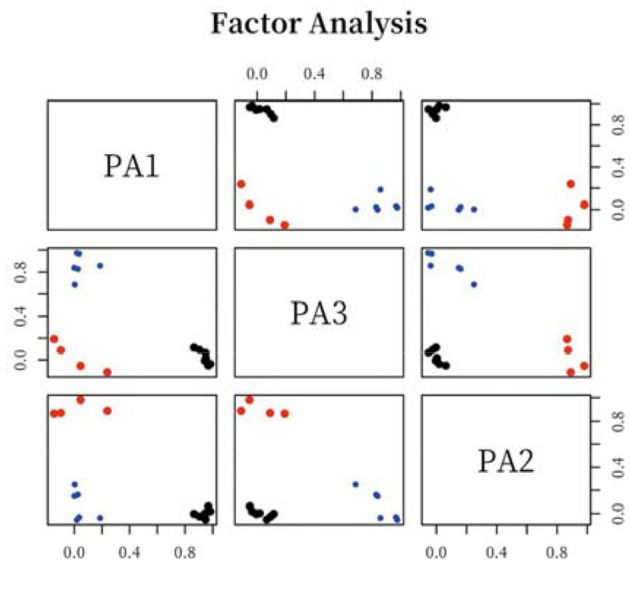
Distribution of color semantics in the two-dimensional cognitive space.

After determining the three common factors, the system will transform the factor scores of all the samples into a visual scatter diagram, by which we can more intuitively judge and analyze the spatial distribution of color images in beauty packaging. Figure 7 shows the distribution of color semantics in the three-dimensional cognitive space (composed of three factors). Figure 8 shows that the two-dimensional cognitive space matrix is composed of three factors matching with each other. The influence of semantics on public factors can be determined by its position in space, while the distance between them indicates the similarities and differences of semantics. Hence, when evaluating the color image of beauty packaging, the corresponding evaluation semantic system can be selected according to the distribution position and distance of cognitive space.

**Figure 9:**
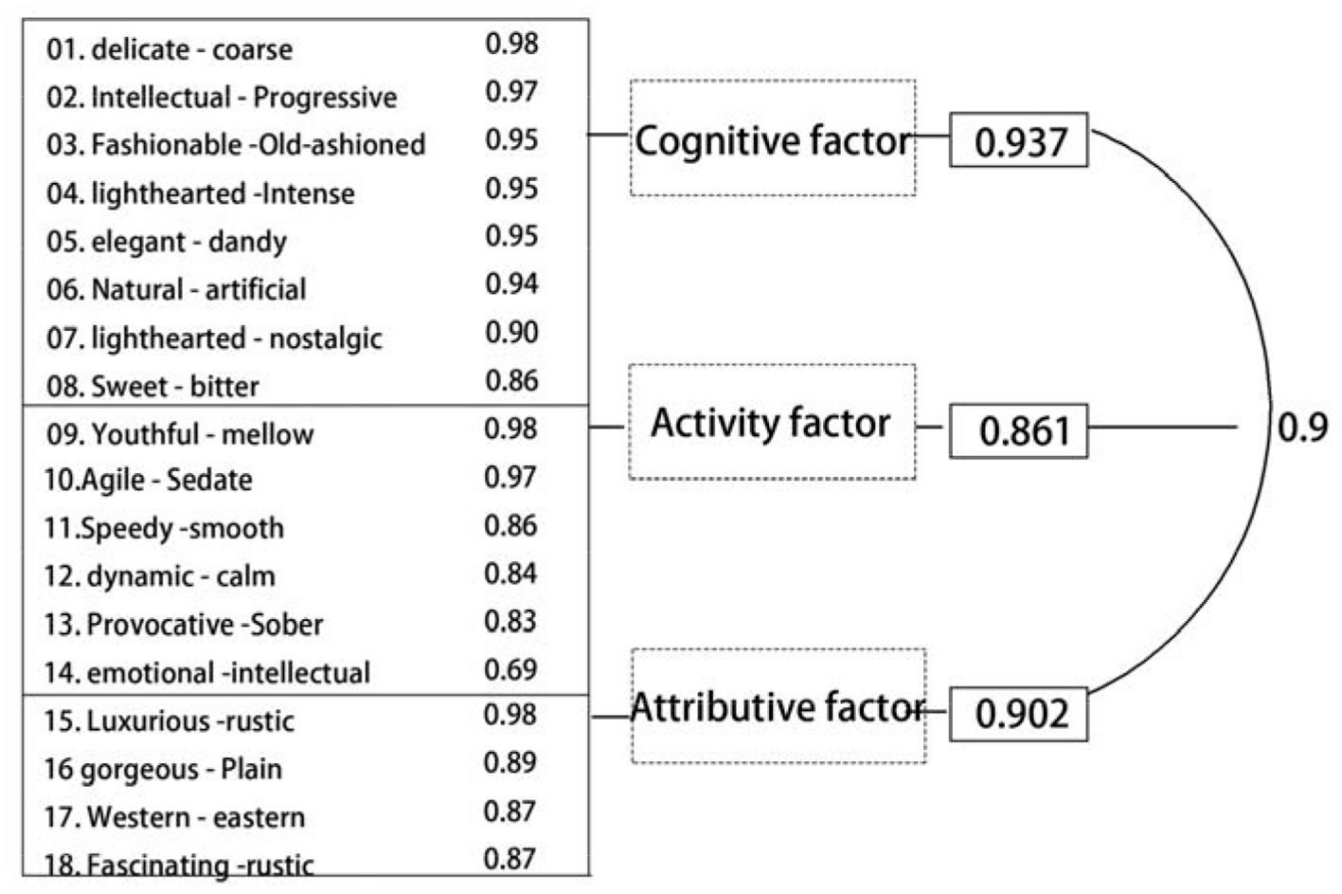
Numerical analysis diagram of three common factors.

Through the comparative analysis of the representative semantics of the three common factors (factor axis) extracted from the factor analysis and the color attributes of the corresponding representative samples, the corresponding relationship and correlation characteristics between the sample color attributes and the semantics conveyed by factor axis are discussed. It can be seen from Figure 9 that the standardization coefficient of each path is < 1, and *P*-values are more than 0.8, indicating that the cognitive attribute, activity attribute, and attribution attribute of cosmetic packaging color will affect the willingness of consumers to decide which beauty packaging to choose, and that all of them will have a significant positive impact. Among them, the cognitive attribute plays the most important role, the attribution attribute of beauty packaging plays a second role in the choices of consumers, and the last is the activity attribute.

## Narrative Analysis of Questionnaire on Beauty Packaging Color

**Figure 10:**
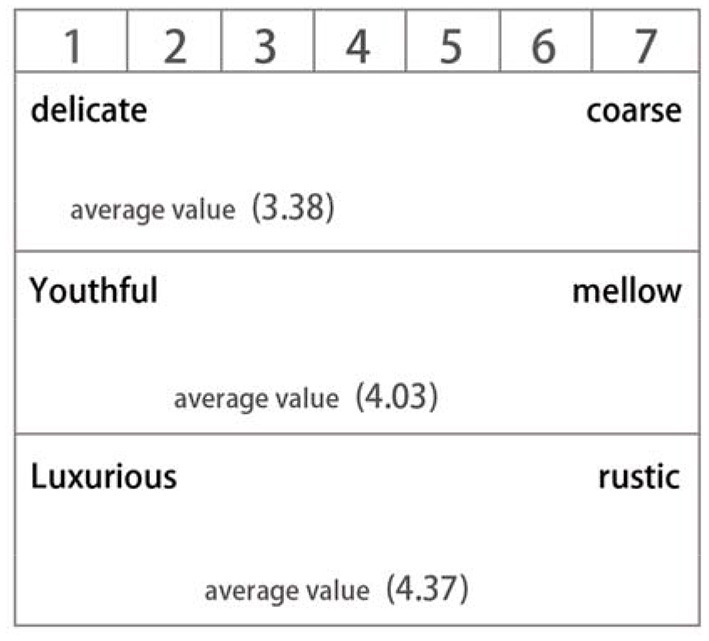
Average value of semantics represented by three common factors.

Based on the color sample statistics of 97 beauty packaging and using a Likert scale, the representative semantics of the three common factors are extracted. Figure 10 shows that the average value of “delicate/coarse” in the “cognitive factor” is 3.38, the average value of “youthful/mellow” in the “activity factor” is 4.03, and the average of “luxurious/rustic” in the “attributive factor” is 4.37. It can be seen that at the level of rational cognition, the group prefers “delicate” to the color tendency of beauty packaging; at the level of experience and feelings, the group prefers “mellow”; and at the level of social geography, the group prefers “rustic.”

### Color Attributes Characteristics of Semantics Represented by Cognitive Factor

The first common factor (cognitive factor) extracted from the factor analysis shows that its representative vocabularies are delicate/coarse, intellectual/progressive, and fashionable/old-fashioned. The most remarkable samples of negative axes (delicate, intellectual, fashionable) on this factor are: YR/B, Y/B, G/L, BG/L, B/L, G/S, BG/S; the most remarkable samples of positive axes (coarse, progressive, and old-fashioned) are R/DGR, YR/DGR, and Y/DGR. By analyzing the color attributes of typical sample colors, we observe that the color features corresponding to approximate sensory images such as delicate, intellectual, and fashionable, are mainly as follow: the color has a visual perception of weak brightness, and the change in chromaticity is also inclined to increase the content of white. In terms of hue, blue-green color is the most remarkable, and there are also some colors of orange and yellow, which show the psychological feelings of visual cognition and prefer soft colors, while the color brightness and purity of main-tune tendency prefer stable color combinations with little fluctuation. It shows that women of this age group with a natural sense of closeness to rural life prefer neutral and cold colors in hue, and turbid colors, which have low color purity and tend to gray system. The women in northern China are reserved, and the colors they prefer have the characteristics of low brightness and low purity, and basically form the conservative and cautious semantics, which is consistent with their personalities.

### Color Attributes Characteristics of Semantics Represented by Activity Factor

The second common factor (activity factor) extracted from the factor analysis shows that its representative vocabularies are youthful/mellow, agile/sedate, and speedy/smooth. The most remarkable samples of negative axes (youthful, agile, speedy) on this factor are: RP/V, R/S, B/S; the most remarkable samples of positive axes (mellow, sedate, smooth) are: PB/B, PB/GR, B/GR. By analyzing the color attributes of typical sample colors, we observe that the color features corresponding to approximate sensory images such as mellow, sedate, and smooth, are mainly as follow: the color has a visual perception of weak brightness, and the change in chromaticity is also inclined to increase the content of black. In terms of hue, blue-purple color is the most remarkable; chromaticity also tends to the middle gray scale of achromatic colors. Colors of gray and blue-gray are the main hues, which show the reserved and conservative characteristics of the group. The tone is mainly cloudy and gray, which is closer to the quiet and calm inner feelings, contrary to the semantic characteristics such as being open and enthusiastic.

### Color Attributes Characteristics of Semantics Represented by Attributive Factor

The third common factor (attributive factor) extracted from the factor analysis shows that its representative vocabularies are luxurious/rustic, gorgeous/plain, and western/eastern. The most remarkable samples of negative axes (luxurious, gorgeous, western) on this factor are R/DP and YR/DP; the most remarkable samples of positive axes (plain, eastern, rustic) are GY/Lgr, G/Lgr, BG/Lgr, P/P, and N8. By analyzing the color attributes of typical sample colors, we observe that the color features corresponding to approximate sensory images such as plain, eastern, and rustic, are mainly as follow: in terms of brightness, it is better to choose middle brightness, and the change in chromaticity is also inclined to increase the content of white. In terms of hue, the colors of blue, purple, and gray, which can provide the best visual experience by reflecting the oriental life, also reflect the color emotional experience of the group in the specific cultural background.

## Conclusion

Nowadays, perceptual engineering and color psychology are both emerging fields in the design industry. They explore the relationship between rational thinking and perceptual impression of humans, how aesthetic experience of people is formed, and how to construct the relationship between human emotions, environment, and objects. A good communication bridge between the participants and finding effective emotional design methods are issues worthy of further investigation. NCD color image positioning, as the front-end system of color design, is the scientific color theory basis for fashion product designers. This study constructs a complete color reference system by investigating and positioning the color image of middle-aged women in the rural areas of northern China, focusing on exploring the co-existence of the color design process on color, women, and the environment. The proposed color-oriented design method conforms to the development trend of scientific design, clarifies the color logic relationship of “color impression and emotional semantics,” and is oriented to enhance the emotional experience of the user, which is conducive to accurately creating aesthetic characteristics of the local market. The product color with cultural connotation highlights the application value. At the same time, the construction of the color system meets the aesthetic needs of the Chinese population, and has the commercial practice value of more optimized design and more scientific structure.

## Data Availability Statement

Publicly available datasets were analyzed in this study. This data can be found here: www.wjx.cn, https://www.wjx.cn/mobile/statnew.aspx?activity=111860856&reportid=#1, https://www.wjx.cn/vm/tU4Ai4A.aspx.

## Author Contributions

All authors listed have made a substantial, direct and intellectual contribution to the work, and approved it for publication.

## Conflict of Interest

The authors declare that the research was conducted in the absence of any commercial or financial relationships that could be construed as a potential conflict of interest.

## Publisher's Note

All claims expressed in this article are solely those of the authors and do not necessarily represent those of their affiliated organizations, or those of the publisher, the editors and the reviewers. Any product that may be evaluated in this article, or claim that may be made by its manufacturer, is not guaranteed or endorsed by the publisher.
